# MicroRNA-381 Regulates Chondrocyte Hypertrophy by Inhibiting Histone Deacetylase 4 Expression

**DOI:** 10.3390/ijms17091377

**Published:** 2016-08-23

**Authors:** Weishen Chen, Puyi Sheng, Zhiyu Huang, Fangang Meng, Yan Kang, Guangxin Huang, Zhiqi Zhang, Weiming Liao, Ziji Zhang

**Affiliations:** Department of Joint Surgery, First Affiliated Hospital of Sun Yat-sen University, Guangzhou 510080, China; chenweishen5@163.com (W.C.); shengpuyi@hotmail.com (P.S.); wongchiyue@163.com (Z.H.); fanfan71@126.com (F.M.); neokang@163.com (Y.K.); huangguang06@163.com (G.H.); zhzhiqi@163.com (Z.Z.)

**Keywords:** microRNA-381, chondrocyte hypertrophy, histone deacetylase 4, Runt-related transcription factor 2, matrix metalloproteinase 13

## Abstract

Chondrocyte hypertrophy, regulated by Runt-related transcription factor 2 (RUNX2) and matrix metalloproteinase 13 (MMP13), is a crucial step in cartilage degeneration and osteoarthritis (OA) pathogenesis. We previously demonstrated that microRNA-381 (miR-381) promotes MMP13 expression during chondrogenesis and contributes to cartilage degeneration; however, the mechanism underlying this process remained unclear. In this study, we observed divergent expression of miR-381 and histone deacetylase 4 (HDAC4), an enzyme that directly inhibits RUNX2 and MMP13 expression, during late-stage chondrogenesis of ATDC5 cells, as well as in prehypertrophic and hypertrophic chondrocytes during long bone development in E16.5 mouse embryos. We therefore investigated whether this miRNA regulates HDAC4 expression during chondrogenesis. Notably, overexpression of miR-381 inhibited HDAC4 expression but promoted RUNX2 expression. Moreover, transfection of SW1353 cells with an miR-381 mimic suppressed the activity of a reporter construct containing the 3′-untranslated region (3′-UTR) of HDAC4. Conversely, treatment with a miR-381 inhibitor yielded increased HDAC4 expression and decreased RUNX2 expression. Lastly, knockdown of HDAC4 expression resulted in increased RUNX2 and MMP13 expression in SW1353 cells. Collectively, our results indicate that miR-381 epigenetically regulates MMP13 and RUNX2 expression via targeting of HDAC4, thereby suggesting the possibilities of inhibiting miR-381 to control chondrocyte hypertrophy and cartilage degeneration.

## 1. Introduction

Osteoarthritis (OA), the most common disabling joint disease world-wide, is characterized by articular cartilage degeneration and subchondral bone remodeling. However, the underlying etiology and pathogenesis of this disease are poorly understood [1,2]. It is widely accepted that the matrix metalloproteinase (MMP) family plays a pivotal role in cartilage degradation and OA pathogenesis by promoting the degradation of the extracellular cartilage matrix (ECM), thereby leading to cartilage loss [2,3,4,5,6]. Among the MMP family, MMP13 (also known as Collagenase-3) exhibits the strongest collagenolytic activity leading to cartilage degeneration in OA patients [3,4,7]. Meanwhile, Runt-related transcription factor 2 (RUNX2), a member of the RUNX family that promotes chondrocyte hypertrophy, endochondral ossification, and skeletal development [8,9], was shown to enhance *MMP13* promoter activity and thereby promote cartilage degeneration [10,11].

Histone deacetylases (HDACs) are a vast family of enzymes that repress the transcriptional activity of certain genes by condensing the surrounding chromatin [12]. Recently, several HDACs were identified as important regulators of cartilage development and degradation [13,14,15,16]. In particular, HDAC1 and HDAC2 were shown to repress cartilage-specific gene expression [14], and HDAC7 was found to suppress chondrocyte proliferation and β-catenin activity [16]. Meanwhile, HDAC4 was shown to inhibit chondrocyte hypertrophy and endochondral bone formation by directly interacting with and inactivating RUNX2 [13]. HDAC4 was also shown to inhibit *RUNX2* and *MMP13* promoter activity [17]. Notably, HDAC4-null mice display early onset chondrocyte hypertrophy and subsequent premature ossification [13], and decreased expression of HDAC4 was found to contribute to the pathogenesis of cartilage degeneration in OA [17]. Together, these findings indicate that HDAC4 is a key regulator of chondrocyte hypertrophy and skeletal development [13].

MicroRNAs (miRNAs, miR) are endogenous ~22 nt non-coding small RNAs that function as important post-transcriptional regulators. miRNAs mediate gene silencing by binding to the 3′-untranslated region (3′-UTR) of the target mRNA, inducing its degradation [18,19]. As an important epigenetic regulator, HDAC4 was found to be a crucial target of microRNAs in several diseases and cell types. For example, miR-29b specifically targets HDAC4 to epigenetically regulate multiple myeloma cell growth and survival [1], miR-22 targets and inhibits HDAC4 in antigen-presenting cells and plays a critical role in emphysema and TH17 responses [7], miR-125a-5p targets HDAC4 to suppress breast tumorigenesis [3], and miR-140 and miR-365 target HDAC4 and participate in cartilage and bone development [20,21].

Several miRNAs have been shown to play important roles in chondrogenesis and cartilage degeneration [22]. Using a miRNA microarray technique, we previously identified 12 miRNAs that were differentially regulated during chondrogenic induction of human adipose-derived stem cells. Of these, eight (miR-193b, miR-199a-3p/hsa-miR-199b-3p, miR-455-3p, miR-210, miR-381 (also known as miR-381-3p), miR-92a, miR-320c, and miR-136) were upregulated, while the other four (miR-490-5p, miR-4287, miR-BART8*, and miR-US25-1*) were downregulated during this process [22]. Based on these findings, we investigated the possible mechanisms by which these miRNAs regulate cartilage development and degradation. We demonstrated that miR-193b regulates early chondrogenesis by targeting transforming growth factor β 2 (TGFB2) and TGFBR3 [23], and that miR-455-3p might promote early chondrogenesis by targeting RUNX2 [24]. Furthermore, miR-320 was found to target MMP13, and the downregulation of this miRNA was predicted to contribute to OA pathogenesis [5]. In a previous study, we reported a 3.15-fold increase in miR-381 expression upon differentiation of human adipose-derived stem cells into chondrocytes [22]. We also noted that miR-381 is highly expressed in osteoarthritic chondrocytes in OA patients as well as in interleukin-1β-treated chondrocytes [25]. While miR-381 was observed to play an important role in cartilage degradation by enhancing MMP13 expression and repressing type II collagen (COL2A1) expression, we were unable to identify the target of this miRNA [25]. By using luciferase reporter assay, we demonstrated that neither nuclear factor of kappa light polypeptide gene enhancer in B-cells inhibitor α (NFΚBIA) nor NFKB repressing factor (NKRF) was the target of miR-381 [25]. However, using miRNA target prediction algorithms, we discovered that HDAC4 might be a potential target of miR-381. Since miR-381 and HDAC4 are essential for mediating chondrocyte hypertrophy and cartilage degeneration, we hypothesized that miR-381 participates in OA pathogenesis by targeting and suppressing HDAC4 expression.

## 2. Results

### 2.1. Divergent Expression of MicroRNA-381 (miR-381) and Histone Deacetylase 4 (HDAC4) during Late-Stage Chondrogenesis of ATDC5 Cells

**Figure 1:**
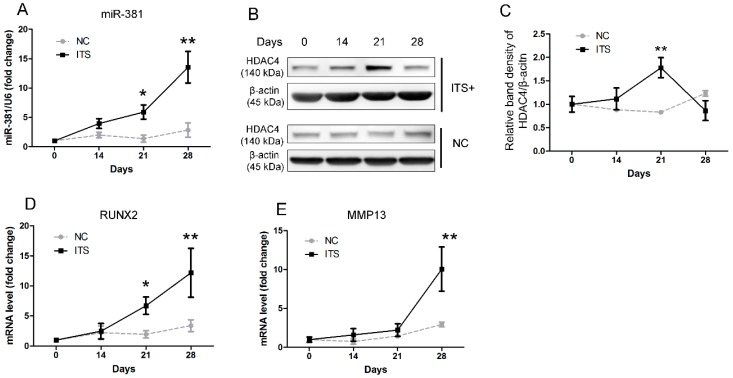
Evaluation of the expression patterns of miR-381 and HDAC4, and of the hypertrophic markers RUNX2 and MMP13 during the chondrogenesis of ATDC5 cells. ATDC5 cells were cultured with/without ITS (insulin (10 μg/mL), transferrin (10 μg/mL), and sodium selenite (3 × 10^−8^ M)) and harvested on the indicated days. (**A**–**E**) Quantitative real-time reverse transcription (qRT)-PCR and Western blot analyses were utilized to evaluate the mRNA expression levels of (**A**) miR-381, (**D**) RUNX2, and (**E**) MMP13, and the protein expression levels of (**B**) HDAC4 during chondrogenesis, respectively; (**C**) graphic depiction of the protein expression levels quantified from the data presented in panel **B**. Data in panels **A**, **C**, **D**, and **E** are presented as means ± standard deviations of the results of three independent experiments. Negative control (NC) indicates cells cultured without ITS. * *p* < 0.05; ** *p* < 0.001. miR-381, microRNA-381; HDAC4, histone deacetylase 4; RUNX2, Runt-related transcription factor 2; MMP13, matrix metalloproteinase 13.

To characterize the expression patterns of miR-381 and HDAC4 during chondrogenesis, ATDC5 cells were cultured in the presence of ITS ((insulin (10 μg/mL), transferrin (10 μg/mL), and sodium selenite (3 × 10^−8^ M)) to induce differentiation into chondrocytes. There was significantly greater expression of miR-381 in cells treated with ITS at 21 and 28 days post-induction than in those cultured in the absence of ITS (Figure 1A). In contrast, HDAC4 expression peaked at day 21 and decreased significantly at day 28 post-induction (Figure 1B,C). Meanwhile, the expression patterns of the two chondrocyte-hypertrophy markers RUNX2 and MMP13 were similar to that of miR-381 during chondrogenesis of ATDC5 cells (Figure 1D,E).

### 2.2. Evaluation of miR-381 and HDAC4 Expression during Cartilage Development

**Figure 2:**
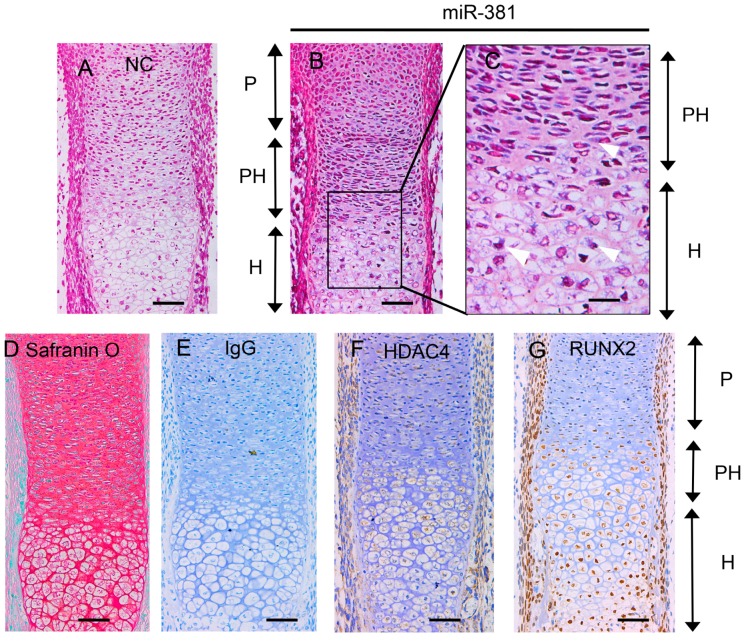
Evaluation of miR-381, HDAC4, and RUNX2 expression in E16.5 mouse radius bones. In situ hybridization analysis of (**A**) the negative control and (**B**) miR-381 expression are shown; (**C**) enlarged view of the boxed area in panel B; white arrows show areas of positive staining for miR-381; (**D**) sections were stained with safranin O/Fast Green for observation of chondrocyte morphology; and (**E**–**G**) sections were subjected to immunohistochemistry (brown) analysis using normal (**E**) IgG (negative control), (**F**) HDAC4-specific, and (**G**) RUNX2-specific antibodies. Scale bar = 20 μm in panel **C** and 50 μm in the other panels. miR-381, microRNA-381; HDAC4, histone deacetylase 4; RUNX2, Runt-related transcription factor 2. P, proliferating chondrocytes; PH, prehypertrophic chondrocytes; H, hypertrophic chondrocytes.

To assess the levels of miR-381 expression during different stages of cartilage development, the forelimbs of mouse embryos at embryonic day 16.5 (E16.5) were subjected to in situ hybridization analysis. While low levels of miR-381 expression were observed in proliferating chondrocytes, expression of this miRNA increased significantly in pre-hypertrophic and hypertrophic chondrocytes (Figure 2A–C). Notably, this expression pattern was similar to that of RUNX2 (Figure 2G). In contrast, HDAC4 expression peaked in pre-hypertrophic chondrocytes and decreased in hypertrophic chondrocytes, as determined by immunohistochemistry analysis (Figure 2D–F). These results are therefore consistent with those obtained using chondrogenic ATDC5 cells.

### 2.3. miR-381 Inhibits the mRNA and Protein Expression of HDAC4 in SW1353 Cells

**Figure 3:**
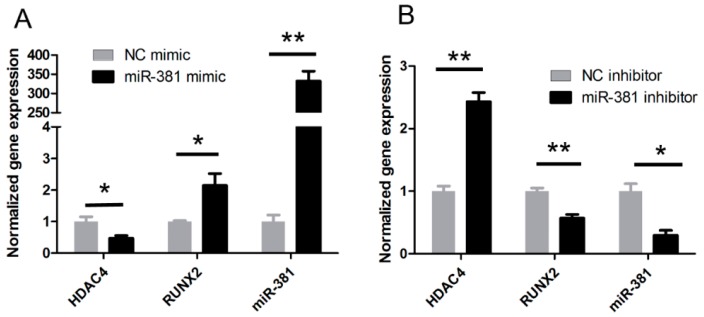

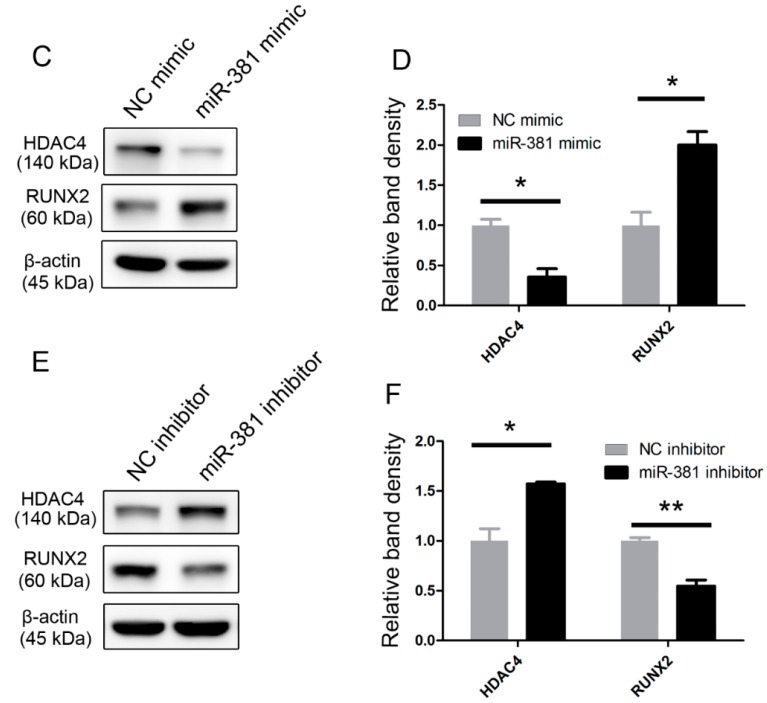
Evaluation of the mRNA and protein expression levels of HDAC4 and RUNX2 after overexpression/knockdown of miR-381. (**A**,**B**) SW1353 cells were transfected with (**A**) a miR-381 mimic or (**B**) a miR-381 inhibitor and the mRNA expression levels of HDAC4 and RUNX2 were determined by quantitative real-time reverse transcription (qRT)-PCR analysis; (**C**–**F**) Western blot analysis of HDAC4 and RUNX2 expression in SW1353 cells transfected with (**C**,**D**) a miR-381 mimic or (**E**,**F**) a miR-381 inhibitor. Panels D and F contain graphic depictions of the protein expression levels quantified from the data presented in panels C and E, respectively. Data are presented as means ± standard deviations from three independent experiments. * *p* < 0.05; ** *p* < 0.001. miR-381, microRNA-381; HDAC4, histone deacetylase 4; RUNX2, Runt-related transcription factor 2.

The divergent expression of miR-381 and HDAC4 observed during late-stage chondrogenesis of ATDC5 cells and in the long bones of mouse embryos implies that miR-381 might directly inhibit HDAC4 expression. To address this hypothesis, HDAC4 mRNA and protein expression levels were evaluated in SW1353 cells transfected with a miR-381 mimic, or a miR-381 inhibitor by quantitative real-time reverse transcription (qRT-PCR) and Western blot analyses, respectively. While cells overexpressing miR-381 exhibited significantly decreased mRNA and protein expression of HDAC4, those treated with the miR-381 inhibitor showed increased HDAC4 expression (Figure 3). Conversely, overexpression and inhibition of miR-381 resulted in markedly increased and decreased RUNX2 mRNA and protein expression, respectively (Figure 3).

### 2.4. Small Interfering RNA (siRNA)-Mediated Knockdown of HDAC4 Promotes the Expression of Runt-Related Transcription Factor (2RUNX2) and Matrix Metalloproteinase 13 (MMP13)

**Figure 4:**
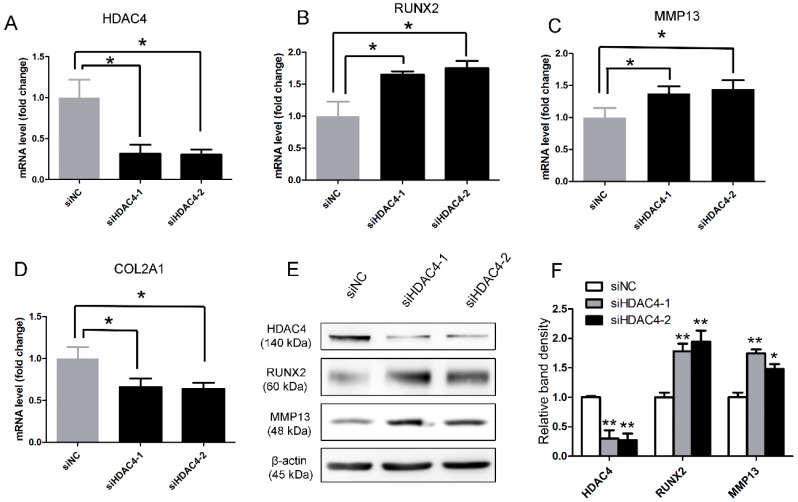
Effects of siRNA-mediated silencing of HDAC4 on RUNX2, MMP13, and COL2A1 expression. (**A**–**D**) SW1353 cells were transfected with two different HDAC4-specific siRNA molecules for 6 h and then cultured for another 24 h. Subsequently, the mRNA expression levels of (**A**) HDAC4, (**B**) RUNX2, (**C**) MMP13, and (**D**) COL2A1 were measured by quantitative real-time reverse transcription (qRT)-PCR analysis; (**E**) SW1353 cells were transfected with the two HDAC4-specific siRNA molecules for 6 h and cultured for another 48 h. Cells were then harvested and subjected to Western blot analysis to evaluate the protein expression levels of HDAC4, RUNX2, MMP13 (active form), and β-actin; (**F**) graphic depiction of the protein expression levels quantified from the data presented in panel E. Data are presented as means ± standard deviations from three independent experiments. * *p* < 0.05; ** *p* < 0.001. miR-381, microRNA-381; HDAC4, histone deacetylase 4; RUNX2, Runt-related transcription factor 2; MMP13, matrix metalloproteinase 13; COL2A1, type II collagen.

To evaluate the effects of HDAC4 on RUNX2 and MMP13 expression, SW1353 cells were transfected with two HDAC4-specific siRNA molecules, siHDAC4-1 and siHDAC4-2, respectively. The depletion efficiencies for siHDAC4-1 and siHDAC4-2 were approximately 70% (Figure 4A), and knockdown of HDAC4 resulted in both increased RUNX2 and MMP13 mRNA expression and decreased COL2A1 mRNA expression (Figure 4B–D). Likewise, depletion of HDAC4 also yielded increased RUNX2 and active MMP13 protein expression (Figure 4E,F). Therefore, the effect of HDAC4 siRNA was, to some extent, similar to that of miR-381. These findings further support the hypothesis that miR-381 represses HDAC4.

### 2.5. miR-381 Directly Targets the 3′-Untranslated Region (3′-UTR) of HDAC4 mRNA

**Figure 5:**
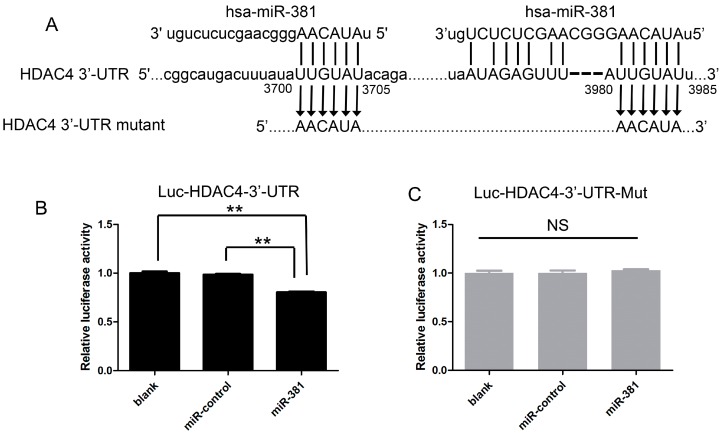
miR-381 modulates HDAC4 expression via interaction with a binding site within the 3′ untranslated region (UTR) of the HDAC4 mRNA; (**A**) sequences of the putative miR-381 binding sites within the 3′-UTR of HDAC4; (**B**,**C**) SW1353 cells were transfected with the wild-type HDAC4 3′-UTR luciferase reporter plasmid (Luc-HDAC4-3′-UTR) alone (blank), or were co-transfected with Luc-HDAC4-3′-UTR or the mutated HDAC4 3′-UTR luciferase reporter plasmid (Luc-HDAC4-3′-UTR-mut) and the miR-381 mimic or nonspecific control microRNA (miR-control). Luciferase activity was then assessed using the Dual-Glo Luciferase Assay System. Data are presented as means ± standard deviations from three independent experiments. ** *p* < 0.001. NS, not significant.

To clarify the molecular mechanism by which miR-381 influences HDAC4 expression, we analyzed the 3′-UTR sequence of the human HDAC4 mRNA using the Targetscan algorithm (Rlease 6.2, Whitehead Institute, Cambridge, MA, USA) and identified a potential miR-381 binding site (UUGUAU) (Figure 5A). Subsequently, to determine whether miR-381 directly modulates HDAC4 expression via interaction with this binding site, we performed luciferase reporter assays using SW1353 cells transfected with vectors harboring the wild-type or mutated 3′-UTR of HDAC4, in the presence or absence of the miR-381 or negative control (NC) mimic. The mutated 3′-UTR sequence was constructed to prevent the binding of the miR-381 mimic (Figure 5A). Compared to cells harboring only the HDAC4 3′-UTR luciferase reporter and cells co-transfected with the reporter vector and the NC mimic, those co-transfected with the reporter vector and the miR-381 mimic exhibited significantly reduced luciferase activity (Figure 5B). In contrast, no significant change in luciferase activity was noted in cells co-transfected with the miR-381 mimic and the luciferase reporter vector containing the mutated 3′-UTR (Figure 5C). These results indicate that miR-381 modulates the expression of HDAC4 by binding to the 3′-UTR of HDAC4 mRNA.

## 3. Discussion

It has been reported that miR-381 has tumor regulatory functions in glioma [26], colorectal cancer [27] and ovarian cancer [28] et al. In addition, miR-381 was found to target IκBα and contributes to respiratory infections [29]. Notably, we demonstrated that miR-381 promotes MMP13 expression and inhibits COL2A1 expression during the chondrogenesis of ATDC5 cells [25]. While we had predicted that miR-381 might target NFΚBIA or NKRF, luciferase reporter assay analyses refuted this hypothesis [25]. In the current study, we observed an inverse expression of miR-381 and HDAC4 during late-stage chondrogenesis of ATDC5 cells as well as in prehypertrophic and hypertrophic chondrocytes during long bone development in E16.5 mouse embryos. In addition, we confirmed that miR-381 targets HDAC4, a key regulator of chondrocyte differentiation [13], and characterized the mechanism by which miR-381 modulates chondrocyte hypertrophy and cartilage degeneration.

Recently, the function of histone-modifying enzymes, including HDACs, has gained intensive interest among researchers [15]. Different classes of HDACs have different effects on chondrocytes. Specifically, Class I HDACs (HDAC1, 2, 3, and 8) are capable of inhibiting the expression of certain cartilage-specific genes, including *COL2A1*, *collagen 9 (α1)*, and *aggrecan* [14]. Additionally, inhibition of Class I HDACs using an HDAC inhibitor or HDAC-specific siRNA molecules resulted in significantly decreased MMP13 expression in interleukin-1 treated chondrocytes [30]. In contrast, HDAC4, a Class II HDAC (HDAC4, 5, 7, 9), was shown to inhibit several chondrocyte hypertrophy/degeneration-related genes, including RUNX2 and MMP13 [13,17]. In mouse primary chondrocytes, adenoviral expression of HDAC4 resulted in reduced acetylation of histone H3 around the *Runx2* promoter, thereby suppressing transcriptional expression of RUNX2 and inhibiting chondrocyte hypertrophy [13]. This effect might be due to the chondroprotective properties of HDAC4. Consistent with these findings, we observed significant increases in the expression of RUNX2 and MMP13 in SW1353 chondrocyte-like cells transfected with HDAC4-specific siRNA molecules. In addition, we observed that HDAC4 is primarily expressed in pre-hypertrophic chondrocytes, exhibiting low levels of expression in hypertrophic chondrocytes from developing long bones of embryonic mice (E16.5). Similarly, HDAC4 expression peaked at 21 days and dropped significantly at 28 days after chondrogenic induction of ATDC5 cells in vitro. Consistent with our findings, HDAC4 was previously shown to be downregulated in the cartilage of OA patients [17]. Therefore, downregulation of HDAC4 during late-stage chondrogenesis might contribute to chondrocyte hypertrophy and cartilage degeneration via the upregulation of RUNX2 and MMP13.

As described above, we previously demonstrated that miR-381 promotes MMP13 expression [25]. In the current study, we noted that RUNX2, which contributes to chondrocyte hypertrophy and cartilage degeneration, was also upregulated by miR-381 at both the mRNA and protein level. Given that the promoter activities of *RUNX2* and *MMP13* are directly inhibited by HDAC4 [17], and that the 3′-UTR of HDAC4 contains potential miR-381 binding sites, we predicted that miR-381 specifically targets and inhibits the expression of HDAC4. Using luciferase reporter assays, we confirmed this conclusion. Meanwhile, qRT-PCR and Western blot analyses supported these data, revealing that miR-381 inhibits HDAC4 mRNA and protein expression in SW1353 cells. Finally, the divergent expression of miR-381 and HDAC4 observed during late-stage chondrogenesis suggests that miR-381 might contribute to the regulation of HDAC4 during the progression from pre-hypertrophic chondrocytes to hypertrophic chondrocytes.

## 4. Materials and Methods

### 4.1. Cell Culture

ATDC5 mouse cells (Riken Cell Bank, Ibaraki, Japan) were cultured in Dulbecco’s Modified Eagle Medium (DMEM) and Nutrient Mixture F-12 (Ham) (Gibco, Grand Island, NY, USA) supplemented with 5% fetal bovine serum (FBS; Gibco), 100 IU/mL penicillin, and 100 µg/mL streptomycin. ATDC5 cells were induced to chondrogenic differentiation using ITS (insulin (10 μg/mL), transferrin (10 μg/mL), and sodium selenite (3 × 10^−8^ M)), as described previously [31,32]. SW1353 human chondrosarcoma cells (American Type Culture Collection, Manassas, VA, USA) were cultured in DMEM/F-12 media supplemented with 10% FBS, 100 IU/mL penicillin, and 100 µg/mL streptomycin. All cells were maintained at 37 °C in a humidified 5% CO_2_ atmosphere.

### 4.2. In Situ Hybridization and Immunohistochemistry

All animal procedures were approved by the ethics committee of the First Affiliated Hospital of Sun Yat-sen University (IRB: 2014C-028, Guangzhou, China). A total number of 6 pregnant C57BL/6J mice (Animal Center of Sun Yat-Sen University, Guangzhou, China) were sacrificed at 16.5 days postcoitum, and the forelimbs of the mouse embryos were dissected and fixed in diethylpyrocarbonate (DEPC)-treated 10% formalin at 4 °C overnight. For miR-381 detection, in situ hybridization was performed using a miR-381-specific probe (Exiqon, Vedbaek, Denmark), as described in our previous studies [24,25].

Immunohistochemistry was performed as described previously [5]. Briefly, samples were fixed in 10% formalin, dehydrated with ethanol, embedded in paraffin, and serially sectioned (5-µm thickness). Sections were then immunostained with antibodies specific to HDAC4 (Santa Cruz Biotechnology, Inc., Dallas, TX, USA) or RUNX2 (Cell Signaling Technology, Danvers, MA, USA). Negative control tissues were stained and incubated with non-immune IgG instead of the specific primary antibodies. Sections were stained with 1% safranin O and 0.5% Fast Green.

### 4.3. Transfection of the miR-381 Mimic, miR-381 Inhibitor, and HDAC4-Specific siRNA Molecules

The miR-381 mimic, miR-381 inhibitor, and HDAC4 siRNA molecules were purchased from RiboBio (Guangzhou, China). Two HDAC4 siRNA molecules were used in this study: HDAC4-1, GCACATATGTACCTAATGA; HDAC4-2, CCATTTCGAATCACTTAAA. Non-specific microRNA mimic, inhibitor, and siRNA molecules were used as negative controls (NC mimic, NC inhibitor, and siNC, respectively). For these analyses, SW1353 cells were seeded into 24-well plates and cultured to 70% confluence. Thereafter, cells were transfected with 100 nM siRNA, 50 nM mimic, or 100 nM inhibitor using Lipofectamine 2000 Transfection Reagent (Invitrogen, Waltham, MA, USA), according to the manufacturer’s instructions. After 6 h, the transfection medium was replaced with DMEM/F-12 supplemented with 10% FBS, and cells were cultured at 37 °C in a humidified 5% CO_2_ atmosphere. Cells were harvested at 24 and 48 h for qRT-PCR assay and Western blot analysis, respectively.

### 4.4. RNA Extraction, Reverse Transcription, and qRT-PCR Analysis

Total RNA was extracted from ATDC5 or SW1353 cells using a miRNeasy Mini Kit (Qiagen, Venlo, The Netherlands). Reverse transcription and qRT-PCR were carried out as described in our previous study [24]. The primer sequences used for these analyses are listed in Table 1. Fold-changes in expression were calculated using the 2^−ΔΔ*C*t^ method [33], and each experiment was performed in triplicate. The expression levels of glyceraldehyde 3-phosphate dehydrogenase (GAPDH) and the small U6 RNA were used as internal controls for mRNA and miRNA, respectively.

### 4.5. Western Blot Analysis

Western blot analysis was performed as previously described [24]. Briefly, total protein was isolated from SW1353 cells, separated by sodium dodecyl sulfate polyacrylamide gel electrophoresis (SDS-PAGE), and transferred to nitrocellulose membranes. Membranes were then incubated with an anti-HDAC4 (Santa Cruz), anti-RUNX2, anti-β-actin (Cell Signaling Technology), or anti-MMP13 antibody (Abcam, Cambridge, UK). β-actin was used as an internal control. Protein bands were visualized using an enhanced chemiluminescence system (GE Healthcare, Little Chalfont, UK), and band densities were analyzed using ImageJ software (National Institutes of Health, Bethesda, MD, USA).

### 4.6. Dual Luciferase Reporter Assay

The region of the HDAC4 3′-UTR containing the predicted seed sequences for miR-381 was PCR amplified using the following primers: Forward, 5′-CGGGCGATCGCTGGAGGTTGCATGGACTGT-3′; Reverse, 5′-AATGCGGCCGCAACACGCTCAGCTTCGTTA-3′. The 3′-UTR fragment was then inserted into the pmiR-RB-REPORT™ luciferase vector (RiboBio) using the SgfI/NotI restriction sites, generating Luc-HDAC4-3′-UTR. Meanwhile, the following primers were used for mutation of two predicted seed sequences within the HDAC4 3′-UTR, generating Luc-HDAC4-3′-UTR-mut: Site 1 forward, 5′-CGGGCGATCGCTGGAGGTTGCATGGACTGTACGACCGGCATGACTTTATAAACATAACAGATTTTGCACGCCAA-3′ and Site 1 reverse 5′-AATGCGGCCGCAACACGCTCAGCTTCGTTA-3′; Site 2 forward, 5′-AGAGTTTAAACATATTATGTGGAAACAGTGTT-3′ and Site 2 reverse, 5′-CCACATAATATGTTTAAACTCTATTATTGGTA-3′.

For luciferase assay analyses, SW1353 cells were seeded into 96-well plates and transfected with 50 nM miR-381 mimic or NC mimic, and 100 ng Luc-HDAC4-3′-UTR or Luc-HDAC4-3′-UTR-mut using Lipofectamine 2000 reagent (Invitrogen), according to the manufacturer’s instructions. The transfected cells were cultured in fresh culture medium for an additional 48 h. Luciferase reporter assays were then performed using the Dual-Glo Luciferase Assay System (Promega, Madison, WI, USA), according to the manufacturer’s instructions.

### 4.7. Statistical Analysis

All statistical analyses were performed using GraphPad Prism software (GraphPad Prism Software, San Diego, CA, USA). Data are presented as means ± standard deviations (SD). All experiments were performed at least three times. Differences between groups were evaluated using Student’s *t*-tests or one-way analysis of variance (ANOVA) with Bonferroni corrected post hoc tests. *p* < 0.05 was considered statistically significant.

## 5. Conclusions

In summary, we demonstrated that miR-381, a crucial microRNA involved in chondrocyte differentiation, directly targets and inhibits the expression of HDAC4, thereby promoting the expression of RUNX2 and MMP13, which, in turn, modulate chondrocyte hypertrophy. Our work therefore indicates that miR-381 comprises a viable target for controlling chondrocyte hypertrophy and cartilage degeneration.

## Figures and Tables

**Table 1:** The primer sequences used in the study.

Genes	Primer Sequences (5′–3′)	
^a^ *mmu/*^b^ *hsa-U6*	Forward	CTCGCTTCGGCAGCACA
	Reverse	AACGCTTCACGAATTTGCGT
*mmu/hsa-miR-381*		TATACAAGGGCAAGCTCTCTGT
*mmu-GAPDH*	Forward	TGTGTCCGTCGTGGATCTGA
	Reverse	TTGCTGTTGAAGTCGCAGGAG
*mmu-RUNX2*	Forward	ATGCTTCATTCGCCTCACAAA
	Reverse	GCACTCACTGACTCGGTTGG
*mmu-MMP13*	Forward	ATGCATTCAGCTATCCTGGCCA
	Reverse	AAGATTGCATTTCTCGGAGCCTG
*hsa-RUNX2*	Forward	CGGAATGCCTCTGCTGTTATG
	Reverse	TTTGTGAAGACGGTTATGGTCAA
*hsa-MMP13*	Forward	GCCAAATTATGGAGGAGATGC
	Reverse	GCCGGTGTAGGTGTAGATAGGAA
*hsa-HDAC4*	Forward	TTTGCCGTGTGTGCTCCATAG
	Reverse	GCGAACAGGCATCAGGTAGGTTA
*hsa-COL2A1*	Forward	GAGGGCAATAGCAGGTTCACGTA
	Reverse	TGGGTGCAATGTCAATGATGG
*hsa-GAPDH*	Forward	ACCCACTCCTCCACCTTTGA
	Reverse	TTGCTGTAGCCAAATTCGTTGT

^a^ mmu indicates mouse; ^b^ hsa indicates human. miR, microRNA; GAPDH, glyceraldehyde 3-phosphate dehydrogenase; RUNX2, Runt-related transcription factor 2; MMP13, matrix metalloproteinase 13; HDAC4, histone deacetylase 4; COL2A1, type II collagen.
